# Comparison of the hemolysis machinery in two evolutionarily distant blood-feeding arthropod vectors of human diseases

**DOI:** 10.1371/journal.pntd.0009151

**Published:** 2021-02-04

**Authors:** Moataza Dorrah, Chaima Bensaoud, Amr A. Mohamed, Daniel Sojka, Taha T. M. Bassal, Michail Kotsyfakis

**Affiliations:** 1 Department of Entomology, Faculty of Science, Cairo University, Giza, Egypt; 2 Institute of Parasitology, Biology Centre, Czech Academy of Sciences, Ceske Budejovice (Budweis), Czech Republic; Faculty of Science, Ain Shams University (ASU), EGYPT

## Abstract

Host blood protein digestion plays a pivotal role in the ontogeny and reproduction of hematophagous vectors. The gut of hematophagous arthropods stores and slowly digests host blood and represents the primary gateway for transmitted pathogens. The initial step in blood degradation is induced lysis of host red blood cells (hemolysis), which releases hemoglobin for subsequent processing by digestive proteolytic enzymes. The activity cycles and characteristics of hemolysis in vectors are poorly understood. Hence, we investigated hemolysis in two evolutionarily distant blood-feeding arthropods: The mosquito *Culex pipiens* and the soft tick *Argas persicus*, both of which are important human and veterinary disease vectors. Hemolysis in both species was cyclical after blood meal ingestion. Maximum digestion occurs under slightly alkaline conditions in females. Hemolytic activity appears to be of lipoid origin in *C*. *pipiens* and enzymatic activity (proteolytic) in *A*. *persicus*. We have assessed the effect of pH, incubation time, and temperature on hemolytic activity and the hemolysin. The susceptibility of red blood cells from different hosts to the hemolysin and the effect of metabolic inhibition of hemolytic activity were assessed. We conclude that in *C*. *pipiens* and *A*. *persicus* midgut hemolysins control the amplitude of blood lysis step to guarantee an efficient blood digestion.

## Author summary

Mosquitoes and ticks are medically important and ubiquitous hematophagous arthropods that require considerable amounts of protein for egg production. Vertebrate blood is the source of protein for adult mosquitoes and ticks to mature eggs. *Culex pipiens* is a vector of the filarial agent *Wuchereria bancrofti* and Rift Valley fever (RVFV), Sindbis (SINV), and West Nile (WNV) viruses. Ticks transmit many diseases to domestic animals and rank second to mosquitoes as vectors of human diseases. However, ticks increase potential health hazards several fold since they act as both vectors and reservoirs of pathogens though transstadial and transovarial disease transmission. Most of the nutritional value of a blood meal is proteinaceous, and the largest quantity of protein is contained inside erythrocytes. The digestive features and utilization of dietary components are similar in mosquitoes and soft ticks, but the underlying mechanisms are poorly understood. This study examines the hemolytic activity, lytic cycles, characteristics, and nature of hemolytic factors in the mosquito *C*. *pipiens* and the fowl tick *A*. *persicus*. The work reveals new insights into the distinct digestive processes in both species, improving our understanding of the basic biology of arthropod vectors.

## Introduction

Hematophagous arthropods transmit numerous pathogens that cause severe diseases in humans and animals [1]. Hematophagy (blood-feeding) is an ancient behavior, having evolved independently more than 20 times among arthropods [2]. Mosquitoes and ticks have been evolving independently for hundreds of millions of years. The earliest divergence of mosquitoes between the lineages leading to Anophelinae and Culicinae occurred about 226 million years ago (mya) [3]. Some researchers argue that ticks most likely originated in the Devonian as much as 400 mya [4,5]. Mosquitoes in the *Culex pipiens* complex thrive in temperate and tropical regions worldwide and serve as efficient vectors of several mosquito-borne pathogens [6]. *Culex* (*Culex*) *pipiens* L., 1758 (Diptera: Culicidae: Culicinae), hereinafter referred to as *Culex pipiens*, is of considerable medical importance as a vector of the filarial agent *Wuchereria bancrofti* and viruses including Rift Valley fever, Sindbis, West Nile, Ntaya, and Tahyna viruses [6,7]. *Argas* (*Persicargas*) *persicus* (Oken, 1818) (Ixodida: Argasidae: Argasinae), hereinafter referred to as *Argas persicus*, is a soft tick and an important poultry ectoparasite that spread globally via human transport [8,9]. *Argas persicus* affects poultry directly through blood feeding, and heavy infestations can lead to irritation, restlessness, and reduced productivity due to severe anemia and indirectly through the transmission of pathogens, especially the agent of avian spirochetosis, *Borrelia anserina* [10]. Soft ticks are important vectors and reservoirs of several zoonotic pathogens [10]. *Argas persicus* is a reservoir of emerging human pathogenic rickettsiae of the spotted fever group like *Rickettsia slovaca* (an agent of tick-borne lymphadenopathy) [11] and *R*. *helvetica* (associated with aneruptive fever and acute perimyocarditis) [12]. *Argas persicus* acts as a potential vector of Kyasanur Forest disease virus (KFDV), a reemerging tick-borne viral hemorrhagic fever [13]. Humans contract infection from the bite of nymphs of the tick. Several human cases of KFDV have been recently reported from India and China [14]. Additionally, it acts as an occasional vector of Crimean-Congo hemorrhagic fever (CCHF), a tick-borne zoonosis that causes sporadic cases and severe outbreaks of acute human disease [10]. This tick also transmits Slovakia virus (SLOV), an ungrouped tick-borne virus [15]. The role of *A*. *persicus* as a pathogen vector of multiple serious pathogens, especially in severe infestations, makes this species a public health concern.

Hematophagous arthropod vectors are obligatory blood feeders and many of them tend to ingest large blood meals to reduce the number of host visits and to ensure a long-term nutrient supply [16]. As arthropod ectoparasites invade a wide range of vertebrate hosts, they have adapted their feeding behavior over evolutionary time to protect against the complex host defense system [2]. Blood can be ingested from the hemorrhagic pools that accumulate in tissues following skin lacerations (pool feeders, such as sand flies and ticks) or directly from a cannulated venule or arteriole (vessel feeders, such as triatomine bugs and mosquitoes) [17]. Arthropod blood digestion mechanisms, although not completely understood, are thought to include complex multienzyme processes that can be influenced by feeding stage, host availability, and the intrinsic digestive compartments of the arthropod [18,19]. In the latter context, in adult mosquitoes, the peritrophic membrane (PM) is produced in direct response to blood feeding (type I PM) [20]. The type I PM of adult mosquitoes play key roles in facilitating digestion and protection from pathogens [20]. It may separate digestive events, retain protease inhibitors in the lumen away from the sites of enzyme activity, influence the rate of digestion, and act as a semipermeable barrier [20,21]. Such roles for the PM can be envisaged in ticks. The cysteine peptidase legumain (*Ir*AE) is localized within the peritrophic matrix, beyond in the digestive vesicles of gut cells of *Ixodes ricinus* [22]. The tick PM may act in limiting persistence of the Lyme disease pathogen *Borrelia burgdorferi* within the vector tick *I*. *scapularis* [23].

Hemoglobin, thrombin, albumin, fibrinogen, and transferrin represent about 95% of blood proteins [16]. Hematophagous arthropods possess a powerful network of proteases to digest blood proteins in their midgut [20]. However, since blood feeding evolved independently in Acari-related chelicerates and blood-feeding insects [2], blood processing mechanisms significantly differ among soft ticks and mosquitoes. Mosquito midgut cells synthesize and secret digestive proteases into the gut lumen. While initial large-protein processing occurs in the neutral milieu of the mosquito gut lumen, the resulting peptides from extracellular digestion are absorbed by gut epithelial cells, where they are processed [16]. By contrast, tick digestion occurs solely inside gut cells, with the gut lumen mainly serving as a long-term reservoir for imbibed blood with virtually no digestive proteases [24]. As blood enters the tick midgut, it passes into a large central chamber and then into one of several pairs of branched blind gut caeca, where blood is stored. The filled caeca take up most of tick body cavity. Subsequently, hemoglobin is released from host red blood cells by hemolysis and the major blood proteins are taken up by the digestive gut (heterophagy) and processed in the acidic vesicles of the gut cell endo-lysosomal system [25]. The two major blood protein components–hemoglobin and serum albumin–follow different trafficking routes during heterophagy: albumin is taken up from the lumen by fluid-phase endocytosis and is moved towards small acidic vesicles, while hemoglobin requires specific recognition by cell surface receptors and is directed to large digestive vesicles [26,27]. Despite these different intracellular routes, the proteolytic systems processing these two major blood proteins appear to be identical [28].

We posed the hypothesis that different hemolytic factors (hemolysins) with distinct mechanisms act in the initial stages of blood cell lysis in the gut of female *C*. *pipiens* and *A*. *persicus*, by analogy with related arthropod vectors. In this paper, we report on the activity cycles of hemolysins in female *C*. *pipiens* and *A*. *persicus* as well as the effects of pH, incubation time, and temperature on hemolytic activity; the nature of the hemolysin; red blood cell (RBC) susceptibility in different hosts to hemolysin action; and the effect of a metabolic inhibitor on hemolytic activity.

## Materials and methods

### Ethical statement

Laboratory animals were treated in accordance with the requirements of the Guide for the Care and Use of Laboratory Animals, 8^th^ Edition 2011 (available at: https://grants.nih.gov/grants/olaw/guide-for-the-care-and-use-of-laboratory-animals.pdf).

### Chemicals

All chemicals and reagents used were of standard analytical grade and purchased from Sigma-Aldrich (Saint Louis, MO, USA) unless otherwise indicated.

### Mosquito and tick colonies

The *C*. *pipiens* colony originated from specimens’ field collected at Al Gabal Al Asfar area, Al Qalyubia Governorate, Egypt and reared for 15 generations prior to initiating the experiments. The colony was established in the laboratory at 27 ± 1°C and 60–70% relative humidity. Adults were kept in wooden cages (20 x 20 x 20 cm). At least 100 males and 100 females were placed together in each cage to ensure successful mating. Mosquitoes were provided with 10% sucrose solution *ad libitum* as a supplementary nutrient. For oviposition, small plastic beakers (150 mL) half-filled with tap water were placed in the adult cages. Eggs were transferred to pans (30 cm diameter) containing tap water and covered with muslin. Larvae were provided autoclaved TetraMin flakes (Tetra GmbH, Melle, Germany) (commercial fish food containing 45% crude protein, 5% crude fat, 7% crude fiber, 8% moisture, and 3% sodium chloride) supplemented with baker’s yeast. Rearing water was changed every 3 ± 1 days to avoid contamination with microflora. The experimental *C*. *pipiens* pass through four larval instars and a pupal stage until molting into imagoes. Pupae were collected daily and placed into small plastic beakers for transfer into the breeding cages for adult emergence. Newly molted adults were used in the experiments.

*Argas persicus* ticks were obtained from a chicken farm at Giza Governorate, Egypt and reared for 8 generations before conducting the experiments. Ticks were kept in plastic containers (20 x 20 cm) lined with filter paper, to which they could cling and deposit excreta. Ticks were kept in an incubator at 28°C and 75% relative humidity. The nymphal stage of the experimental *A*. *persicus* constitutes four instars. Last instars were separated, and the emerged adults were used in experiments. Females lay eggs (100–500 eggs/batch) after each blood meal.

### Arthropod blood feeding

Arthropod feeding was assessed as described previously [29]. Briefly, the *in vitro* feeding apparatus consisted of a feeding chamber and a nutritive medium chamber. The feeding chamber included a hollow glass cylinder (7.3 x 2.5 cm) closed at one end with parafilm-membrane (American Can. Co., New York, NY). The other end was covered with muslin to allow mosquito feeding. The nutritive medium chamber consisted of a thick-walled glass container with a bottom partly lined with capillary tubes to prevent its adhesion to the feeding chamber parafilm surface. The apparatus was held at a constant temperature of 37 ± 1°C on a hot plate. The feeding apparatus was kept in the dark to avoid light interruption during feeding. Pigeons and chickens were used as laboratory hosts for mosquitoes and ticks, respectively. Blood feeding duration of *C*. *pipiens* females was around 2–2.5 h. The feeding period of the nymphal *A*. *persicus* ranged from 15–60 min and spanned 30 min to 2 h in the females. Blood used in the *in vitro* feeding system was collected from pigeons, chickens, humans, and sheep in heparinized tubes (3 mL) to prevent clotting.

### Sample preparation

To determine hemolytic activity during the period after blood meal ingestion (during the first or second gonotrophic cycles), adult female mosquitoes and ticks were immobilized in beakers immersed in ice. Groups of 20 cohort females were used per experimental condition for each analysis. They were homogenized with a Teflon homogenizer in phosphate-buffered saline (PBS), pH 7.2 (0.07 M Na_2_HPO_4_, 0.15 M NaCl and 0.45 M CaCl_2_). Homogenates were centrifuged at 1,500 x *g* at 4°C obtained daily for 4 days (mosquitoes) or 30 days (ticks). Supernatants containing the hemolysin were used in the experiments. The total protein concentration in the homogenates was determined using the Total Protein Kit, Micro Lowry (Sigma-Aldrich) with bovine serum albumin as a standard [30].

### Hemolytic activity assay

Host blood was obtained via heparinized syringes (3 mL) to prevent clotting [31]. Heparinized blood was immediately centrifuged at 1500 x *g* and the supernatant removed. RBCs were washed at least three times in isotonic PBS, pH 7.2. RBCs were suspended in 30 volumes of isotonic buffer. A 5 mL RBCs suspension was centrifuged at 1500 x *g* for 3 minutes and the supernatant discarded. RBCs were resuspended in a small amount of isotonic buffer, and 50 μL of arthropod homogenate containing hemolysin solution was added. The solution was diluted to a final volume of 5 mL using isotonic buffer. Tubes containing this mixture were placed in a shaking water bath at 30°C for 180 min. At 60 min intervals, 1 mL aliquots were removed, centrifuged at 2500 x *g* for 3 min, and 0.1 mL of the supernatant was diluted with 5.0 mL isotonic buffer. The absorbance of this solution was measured at 418 nm. RBCs obtained from the zero-time aliquot were fully hemolyzed by the addition of distilled water, centrifuged, and the supernatant was used as a reference to 100% hemolysis to determine the percentage hemolysis of the homogenates. All assays were performed in triplicate. RBCs suspended in buffer were used as a control.

### Homogenate delipidation and hemolytic activity assays

Lipids were extracted from homogenates according to Folch et al. [32], with slight modifications. One mL aliquots of homogenate were added to a 3 mL mixture of redistilled chloroform and methanol (2, 1) (v/v), shaken for 5 min, and incubated until the two layers separated completely. The chloroform layer was removed and filtered through fat-free filter paper. The aqueous phase was rinsed three times with the chloroform-methanol mixture, and the chloroform layer was added to the previous chloroform-lipid extract. The latter was evaporated to dryness in a water bath at 40°C under a stream of nitrogen. Hemolytic activities of the lipid extract and delipidated homogenates were determined.

### Thin-layer chromatography (TLC)

To separate lipid classes, 50 g of silica gel G was thoroughly mixed with 100 mL distilled water and the formed slurry was spread to 0.5 mm thickness on clean glass plates (20 x 20 cm) using an adjustable-thickness spreader. The TLC plate was dried overnight. Before use, each plate was divided with a pin into equal lanes (1.5 cm width), and the chromatogram was washed with the developing solvent until the solvent front reached the top. The chromatogram was air dried, and the lipid samples were applied. Lipid class standards were applied in alternative lanes. The chromatogram was developed with petroleum-ether (boiling point 60–70°C), diethyl ether, and glacial acetic acid in chambers lined with filter paper. The developing solvent was allowed to run until it reached 17 cm from the origin. The chromatogram was removed and air dried and exposed to iodine vapor for visualization of the resolved lipid classes. The lipids were extracted from the gel, and the hemolytic activity of each class was determined.

### The pH and temperature variations during hemolytic activity and effect of metabolic inhibitor

The effect of temperature on hemolytic activity was investigated by running activity assays at 5, 20, 30, 40, and 50°C. The thermal stability of hemolysin at 60°C in both arthropods was evaluated by pre-incubating the homogenate at 60°C for 1 h. The hemolytic activity was then estimated over a pH range (6–8.4 with 0.4 step increase). Both arthropods were fed artificially on a blood meal mixed with 100 μg/mL cycloheximide (a specific inhibitor of cytoplasmic protein synthesis). Hemolytic activities were then measured at different intervals after blood meal ingestion.

### Proteolytic activities using azocasein as a protein substrate

Proteolytic activity was assessed by the release of dye-containing peptides soluble in 8% trichloroacetic acid (TCA), with modifications. The assay reaction mixture contained 0.1 M of the appropriate buffer, 6 mg azocasein, and 0.3 mL of female mosquito homogenate or 0.1 mL of female tick homogenate in a total volume of 1 mL and incubated for 4 h at 30°C. Then, 2 mL of 8% (w/v) TCA was added, and the mixture was centrifuged at 5000 x *g* for 20 min. TCA color-soluble peptides were developed by the addition of 0.5 mL of 2.5 N NaOH. Optical density was measured at 428 nm.

### Acidic proteinase activity determination using either acid- or urea-denatured hemoglobin as a substrate

A denatured hemoglobin solution 2% (w/v) was prepared by placing 2 g of bovine hemoglobin into 100 mL of 0.06 M HCl and centrifugation at 5000 x *g* for 10 min at 3–7°C. Urea-denatured hemoglobin was prepared by dissolving 0.5 g of bovine hemoglobin in 12.5 mL distilled water. Urea (5 g) was added to the hemoglobin solution, followed by 2 mL of 1 M NaOH [33]. The volume was made to 20 mL with distilled water. After 30 min incubation at room temperature, 2.5 mL of 1 M boric acid (prepared in 0.05 M NaCl and 1.1 mL of 0.5 M CaCl_2_) were added, and the final pH was adjusted to 7.5 with 1 M NaCl. The total volume was made to 25 mL with distilled water. The solution was centrifuged at 5000 x *g* for 15 min at 3–7°C, and the supernatant containing denatured hemoglobin was stored at −20°C. Buffers for specific pH ranges were 0.2 M glycine-HCl (pH 2.2–3.6); 0.1 M citric acid, 0.2 M Na_2_HPO_4_ (pH 2.6–7.6); and 0.1 M Tris (pH 7.6–10.0).

Assay reaction mixtures in 1 mL final volume contained 0.1 M buffer to obtain pH values suitable for enzyme dilution. The reaction mixture was incubated at 37°C for 4 h and terminated by addition of 1 mL 5% (w/v) TCA. The precipitate formed was removed by centrifugation at 5000 x *g* for 15 min. The absorbance of the TCA-soluble peptides was determined at 280 nm. Control reaction mixtures without substrate or enzyme were incubated under the same conditions, and the enzyme or substrate was added to the incubation medium after the addition of TCA.

### Alkaline protease activity

Bovine hemoglobin (0.3 mL, 2%) dissolved in 0.15 M NaCl was combined with 0.3 mL of 0.2 M of the pH-appropriate buffer (pH 7–8.6) as a substrate reaction medium. The reaction was initiated by adding the homogenate and incubated at 30°C for 30 min. The reaction was stopped by adding 0.6 mL 20% (w/v) TCA. The supernatant absorbance after centrifugation at 1000 x *g* at 4°C for 10 min was corrected against controls.

### Effect of pH on proteolytic activity

The optimal pH values for general and specific proteolytic activities were identified by running reactions at a pH range from 2 to 10 using the appropriate buffer systems and measuring the activity at each pH as described before.

### Statistical analysis

Data were analyzed with two-way analysis of variance (ANOVA) followed by Šidák post hoc test, after checking for normality with Shapiro-Wilk test (all were distributed normally). In all cases, *p* < 0.05 were considered significant, unless otherwise stated. Statistical analysis was performed with IBM-SPSS Statistics v.25 (IBM, Armonk, NY, USA). Data were expressed as means ± S.E. Each data point represents the mean of three independent replicates (n = 3).

## Results and discussion

### Hemolytic activity in *C*. *pipiens* and *A*. *persicus*

**Fig 1:**
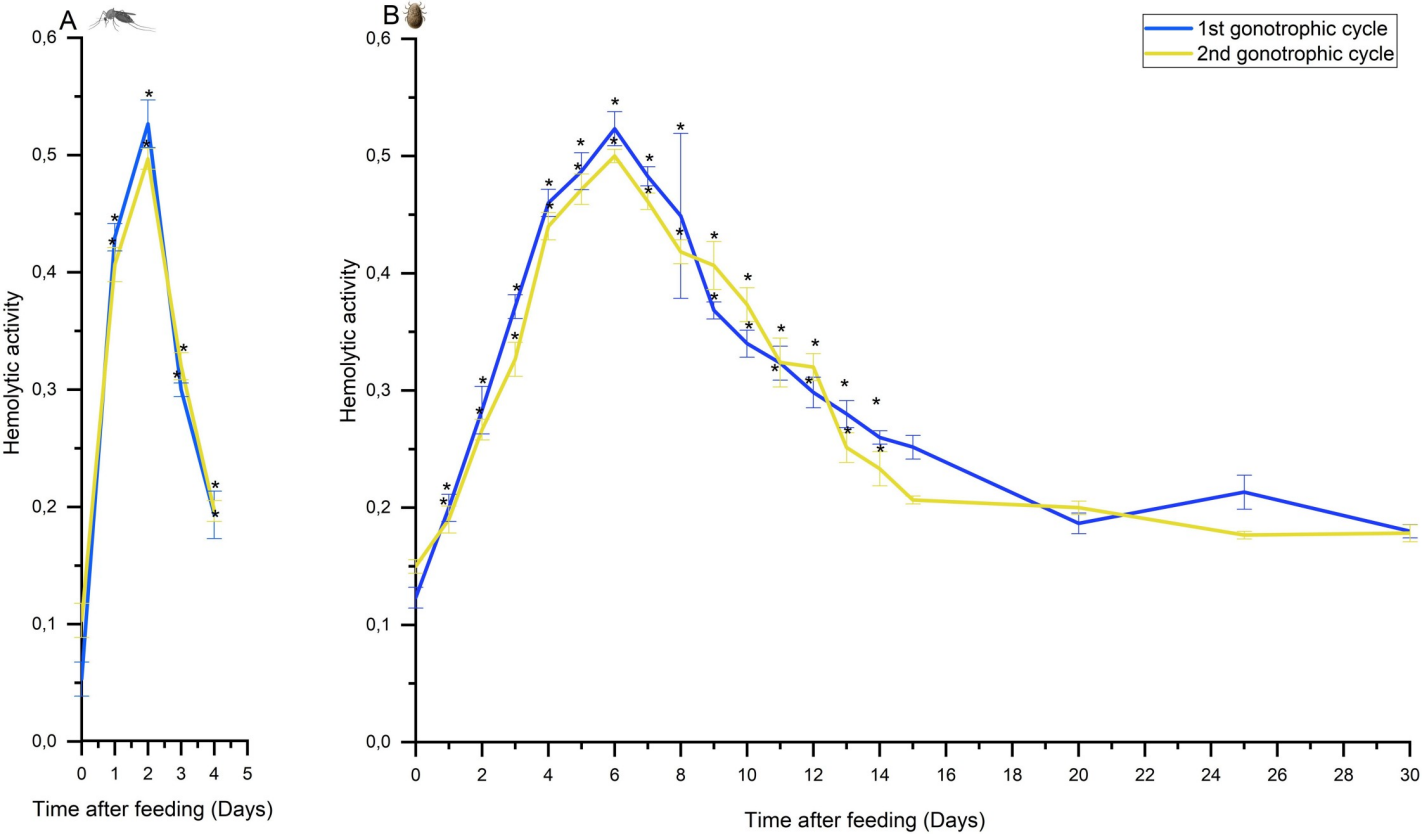
Hemolytic activity in female *Culex pipiens* (A) and *Argas persicus* (B) during the first and second gonotrophic cycles at different intervals after ingestion of pigeon and chicken blood, respectively. Hemolytic activity (± S.E.) is expressed as absorbance at 418 nm/2 h/mg protein (n = 3). *:represent significant difference (*p* < 0.001).

We identified a cyclical pattern in hemolysis during the first and second gonotrophic cycles of *C*. *pipiens* (Fig 1A). During the activity cycle, the hemolysis rate increased sharply after ingestion of the blood meal, reaching a maximum (5–5.8-times the 0 h value) after 48 h of blood meal ingestion. The hemolysis rate subsequently declined to approximately the baseline value by the beginning of the next gonotrophic cycle.

Mosquito and tick gut epithelial histological responses vary considerably during hemolysis. In a related *Culex* mosquito, *C*. *tarsalis*, the period 4–8 h after a blood meal is considered a pre-secreting synthetic period. During this period, the subcellular machinery is synthesized, and the true secretory phase occurs 8–30 h after blood meal ingestion. The secretory phase overlaps significantly with the digestive/absorptive phase, which commences ca. 16 h after feeding and completes after 48 hours, in accordance with our results. After that, the blood meal remnants are excreted, and the gut epithelium enters a cellular resting stage before engorgement on a second blood meal to start a new gonotrophic cycle [34]. Although spread over a longer time course, a pattern of hemolytic activity was observed in *A*. *persicus* to *C*. *pipiens* (Fig 1B) similar to that in another mosquito, *C*. *tarsalis*, despite the different nutritive and digestive strategies used in this tick to mosquitoes. The maximum hemolytic activity in *A*. *persicus* (3.3–4.3-times the 0 h value) was observed at about the middle of the gonotrophic cycle (6 days) and declined thereafter to a low value at the end of this cycle. This latter value, which continued throughout the 30 days of observation, is a maintenance-level value (Fig 1B). Consistent with our findings, the hemolytic activity in related *Argas* (*P*.) *arboreus* [35] also peaked at about day 6 after tick feeding. The hemoglobin in blood meal erythrocytes supplies the bulk of the nutritive protein requirements for mosquitoes [16]. In ticks, hemoglobin appears to be disposable as the amino acid source but instead is a mandatory source of heme in tick reproduction [36]; nevertheless, hemoglobin is an essential nutrient in both animals. As such, prior to its processing in both species, hemoglobin must be released into the gut lumen from the host erythrocyte stroma by hemolysis [19,37]. Generally, in soft ticks, part of the ingested blood meal is retained inside the gut lumen as a food reserve [19], where the majority of hemoglobin undergoes protein crystallization to produce large hemoglobin crystals in the tick gut lumen [38].

The hemolysin(s) in *C*. *pipiens* and *A*. *persicus* were of gut origin, since hemolytic activities were stimulated and increased several times due to the presence of a blood meal in the gut lumen. The salivary gland origin of hemolysin was not considered by Coluzzi et al. [39] and Ribeiro et al. [40], who demonstrated that mosquito and tick salivary glands have no hemolytic activity, respectively. The cyclical intestinal hemolytic activity in both ticks and mosquitoes is most likely controlled by secretagogue mechanisms of gut epithelial cells, which are directly or indirectly stimulated by blood meal components. In comparison, analogous hemolytic activity patterns have been observed in the tsetse fly *Glossina morsitans* [41], *Aedes aegypti* [42], *Stomoxys calcitrans* [43,44], *Rhodnius prolixus* [45,46], and *Ixodes dammini* [38].

**Fig 2:**
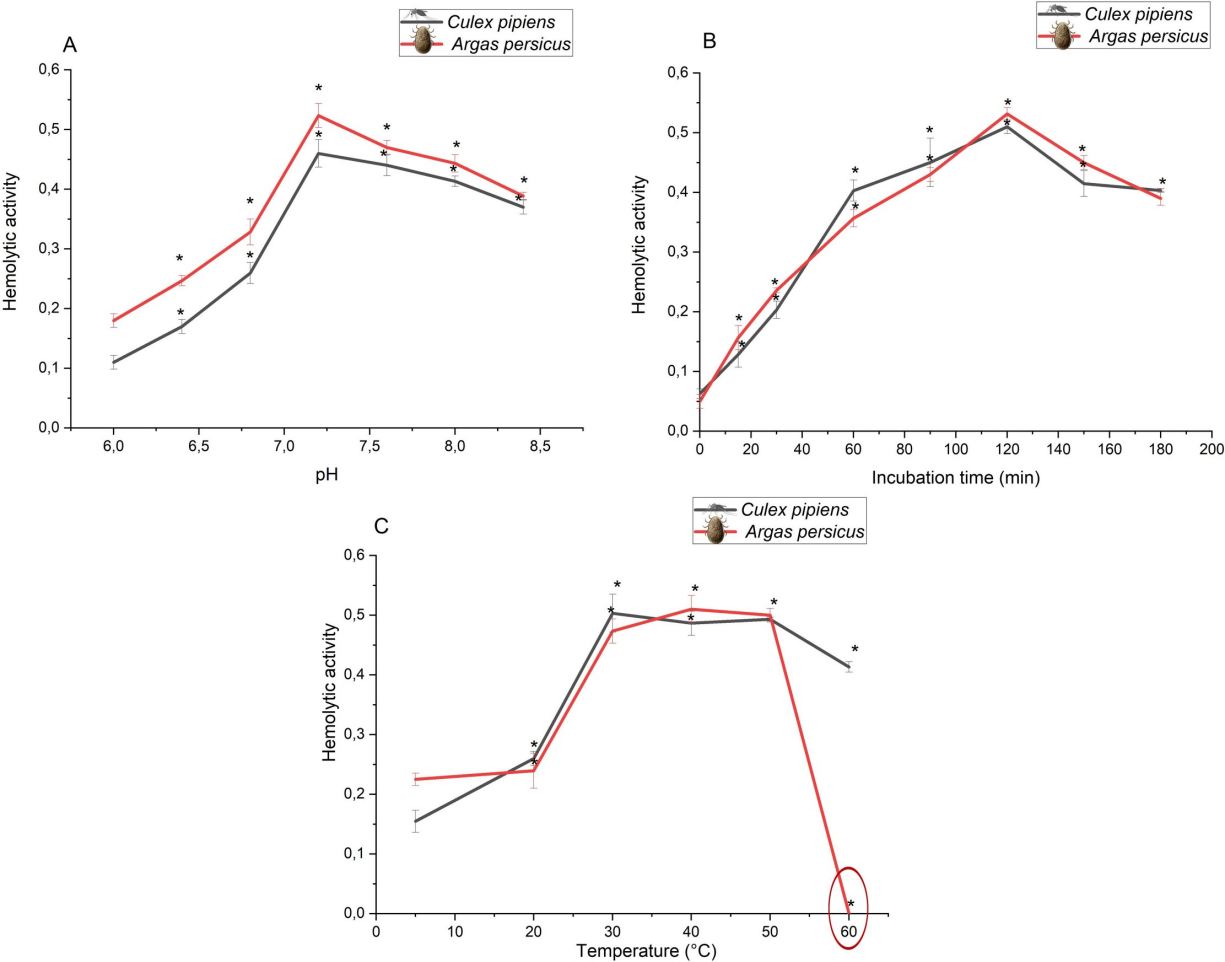
The effect of pH (A), incubation time (B), and temperature (**C**) on hemolytic activity in homogenates of female *Culex pipiens* and *Argas persicus*. Hemolytic activity (±S.E.) is expressed as absorbance at 418 nm/2 h/mg protein and is measured at its maximum (n = 3). *: significantly different (*p* < 0.001).

Hemolysis appears to be induced by specific gut-derived hemolytic factors (hemolysins) and does not appear to be induced by the altered physiological conditions in the gut lumen caused by host erythrocyte rupture. Accordingly, and in order to study, the factors that may affect hemolysis efficiency, we assessed several parameters such as pH, incubation time, and temperature. Testing the influence of pH on hemolytic activity in both species (Fig 2A) confirmed optimal hemolytic conditions at pH 7.2, consistent with the slightly acidic pH in female mosquito gut lumens and a propensity to alkalinity after blood meals [46,47]. Temperature (Fig 2B) had a similar effect on hemolytic activity in both species, with a dramatic decrease in hemolysis in tick samples heated to 60°C for 1 h (protein denaturing conditions). This confirms previous findings [48] that, unlike mosquitoes, host red blood cell membrane removal in the tick midgut is enzymatic. In addition, the maximum hemolytic activity was attained after 120 min of gut sample incubation in reaction medium (Fig 2C) in both *Culex* mosquitoes and *Argas* ticks.

### Nature of the hemolytic factors

**Fig 3:**
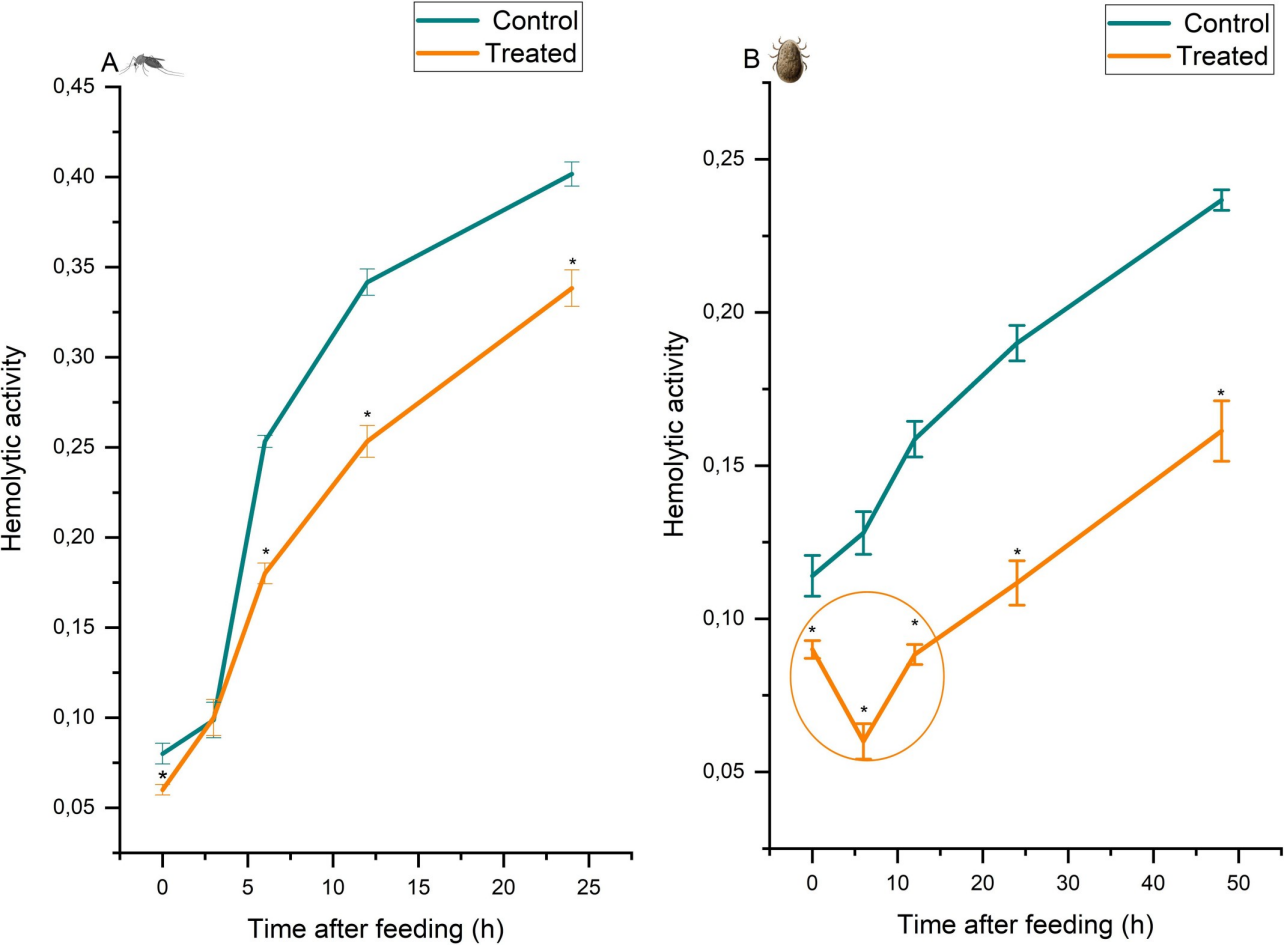
The effect of cycloheximide (100 μg/ mL of blood meal) on hemolytic activity in female *Culex pipiens* (A) and *Argas persicus* (B), after a blood meal. Hemolytic activity (±S.E.) is expressed as the absorbance at 418 nm/2 h/mg protein. Treated (blood + cycloheximide) (n = 3). Data points with the * symbols are significantly different at *p* < 0.001.

Cycloheximide is a naturally occurring fungicide produced by the bacterium *Streptomyces griseus* that inhibits eukaryotic translational elongation in synthesis of a protein; it is widely used as a generic *in vivo* inhibitor of protein synthesis [49]. Here we used it to test the effect of protein synthesis on hemolytic activity to support a role for enzymes indicated by the temperature effect seen in Fig 2B. When applied to *C*. *pipiens* (Fig 3A), cycloheximide only slightly decreased hemolytic activity 5–25 hours after feeding compared to the untreated control group. Therefore, the peptide component of the mosquito hemolytic system appears to contribute little to hemolysis. When applied to *A*. *persicus* (Fig 3B), cycloheximide decreased hemolysis to minimal value after 6 h of feeding, followed by the gradual restoration of hemolytic activity, although still below control values. This pattern indicates translational inhibition of biosynthesized peptides (enzymes) that play a major role in tick hemolytic activity.

Probably due to the independent evolution of blood feeding within several arthropod groups [13], the molecular mechanisms inducing hemolysis are variable in ectoparasites and include the combined action of proteinases and phospholipases [42], lipids [43], and enzymatic polypeptides [37]. Soft ticks have been shown to hemolyze ingested red blood cells [50], but the mechanism remains uncharacterized. We have excluded role of gut microbiota in blood digestion because no bacterial communities were observed in different rearing batches. Midgut microbiota can influence nutrient acquisition by insects [51]. Changing dietary habits of female mosquitoes from nectar as a first meal of high carbohydrate content to the protein-rich blood meal required for their ovarian development strongly modulates the midgut environment, inducing a shift in the midgut microbiome [52,53]. This shift subsequently results in a significant reduction in the overall microbiota diversity, while increased levels of enteric bacteria [53–55]. However, mosquitoes’ digestive tract harbor low bacterial community diversity [56–58], especially in *Culex* spp. where it is dominated by very few bacterial taxa [59,60], that consist mainly of facultative anaerobes and Gram-negative aerobes. Most if not all of these bacteria are acquired from the aquatic habitat the mosquitoes develop in as larvae and transstadially transmitted to the adult, however some but not all bacterial community members are transferred to adults [56,61]. Gut bacteria in adult *A*. *aegypti* may interfere with blood meal digestion [53]. However, gut bacteria were not a source for the digestive proteases. Except the previous work, however, what role, if any, the gut microbiota plays in promoting blood digestion especially the lytic phase of RBCs is unknown in mosquitoes. The ability of certain microbes to facilitate blood digestion in mosquitoes, or the production of products that promote gut function or other activities is currently unknown [62].

Our results (Table 1) demonstrated that the mechanisms inducing hemolysis in *C*. *pipiens* and *A*. *persicus* are different, with 47.1% of total hemolytic activity in *C*. *pipiens* residing in the lipoid fraction and 5.7% in the aqueous fraction in whole-body homogenates. Therefore, the major hemolytic factor in this mosquito is lipoid, with free fatty acids constituting about 71.4% of the hemolytic activity of lipids extracted from whole-body homogenates (Table 1). In *A*. *persicus*, about 60.6% of the total hemolytic activity was in the aqueous fraction and only 1.4% in the lipoid fraction of whole-body homogenates (Table 1), indicating that the major hemolytic factor in this tick species is non-lipoid. This non-lipoid system of hemolysis in *A*. *persicus* is probably peptide-mediated or directly affected by peptides, since (i) the separated hemolytic factor was aqueous; (ii) there was complete inactivation of hemolytic activity by heating at 60°C for 1 h; (iii) it was pH sensitive; and (iv) cycloheximide caused prominent inhibition (Fig 3B).

**Table 1:** Hemolytic activity in female *Culex pipiens* and *Argas persicus* using crude homogenates, organic solvent, and aqueous phases of extraction and the separated lipid classes.

Source	*Culex pipiens*		*Argas persicus*	
	Activity*±S.E.	%Hemolysis	Activity*±S.E.	%Hemolysis^†^
Crude homogenate	0.50±0.012	71.4	0.52±0.015	73.2
Aqueous phase	0.04±0.006	5.7	0.43±0.015	60.6
Organic-solvent phase	0.33±0.015	47.1	0.01±0.003	1.4
Lipid classes				
phospholipid	—		—	
monoglyceride	—		—	
1,2-diglyceride	—		—	
1,3-diglyceride	—		—	
free fatty acids	0.25±0.01	35.7	—	
triglyceride	—		—	
cholesterol ester	—		—	
Control	0.70 ±0.006	100	0.71 ±0.009	100

* = Expressed as absorbance at 418 nm */* 2 h / mg protein.

— = Approximately nil.

^†^ % Hemolysis = activity of sample/activity of control x 100.

The free fatty acid nature of the hemolytic factor in *C*. *pipiens* identified here was consistent with that seen in the stable fly *S*. *calcitrans* [63] and the horn fly *Haematobia irritans* [64]. The observed hemolytic effect of free fatty acids in *C*. *pipiens* might be attributed to their detergent-like character. Cycloheximide, a peptidyl translocase inhibitor, also inhibited hemolytic activity, and certain fatty acids are known to penetrate and eventually (along with other components of the speculated complex lytic system and homogenate content) lyse the erythrocyte membrane according to their structure [65]. The detergent-like effect of free fatty acids may be a prerequisite phase and/or a synergistic component of the peptide system in *C*. *pipiens*. However, a direct or indirect contribution of peptides to hemolysis in *C*. *pipiens* cannot be excluded. Fatty acids are known to produce spiculated erythrocytes [66], a rapid process triggered by the intercalation of the lipophilic portion of fatty acids into the erythrocyte plasma membrane [66]. The detergent-like material not only causes hemolysis at higher concentrations but also supports enzymatic phospholipase attack on membrane phospholipids at sub-lytic concentrations [67]. Hence, we conclude that the detergent-like effect of free fatty acids synergistically acts with phospholipase C and sphingomyelinase to cause erythrocyte hemolysis [44].

Histochemical studies have indicated that glycoprotein hemolysins are present in the gut secretory cells of both the hard tick *Boophilus microplus* and the soft tick *A*. (*P*.) *arboreus* [35]. Peptidic hemolysins have also been proposed in other hematophagous arthropods, for example the mosquito *A*. *aegypti* [42], the tsetse fly *G*. *morsitans* [41], the kissing bug *R*. *prolixus* [45], and the hard tick *I*. *dammini* [68]. The involvement of proteases and/or lipases in hemolysis in hematophagous arthropods is controversial. Geering suggested that proteases and phospholipases were involved in hemolysis in *A*. *aegypti* [42]. However, the involvement of proteases in hemolysis in *G*. *morsitans* [41] and *I*. *dammini* [68] has been excluded. *A*. *persicus* presumably has a non-lipoid hemolysis system, but it may be peptide mediated or directly affected by peptides [44].

**Fig 4:**
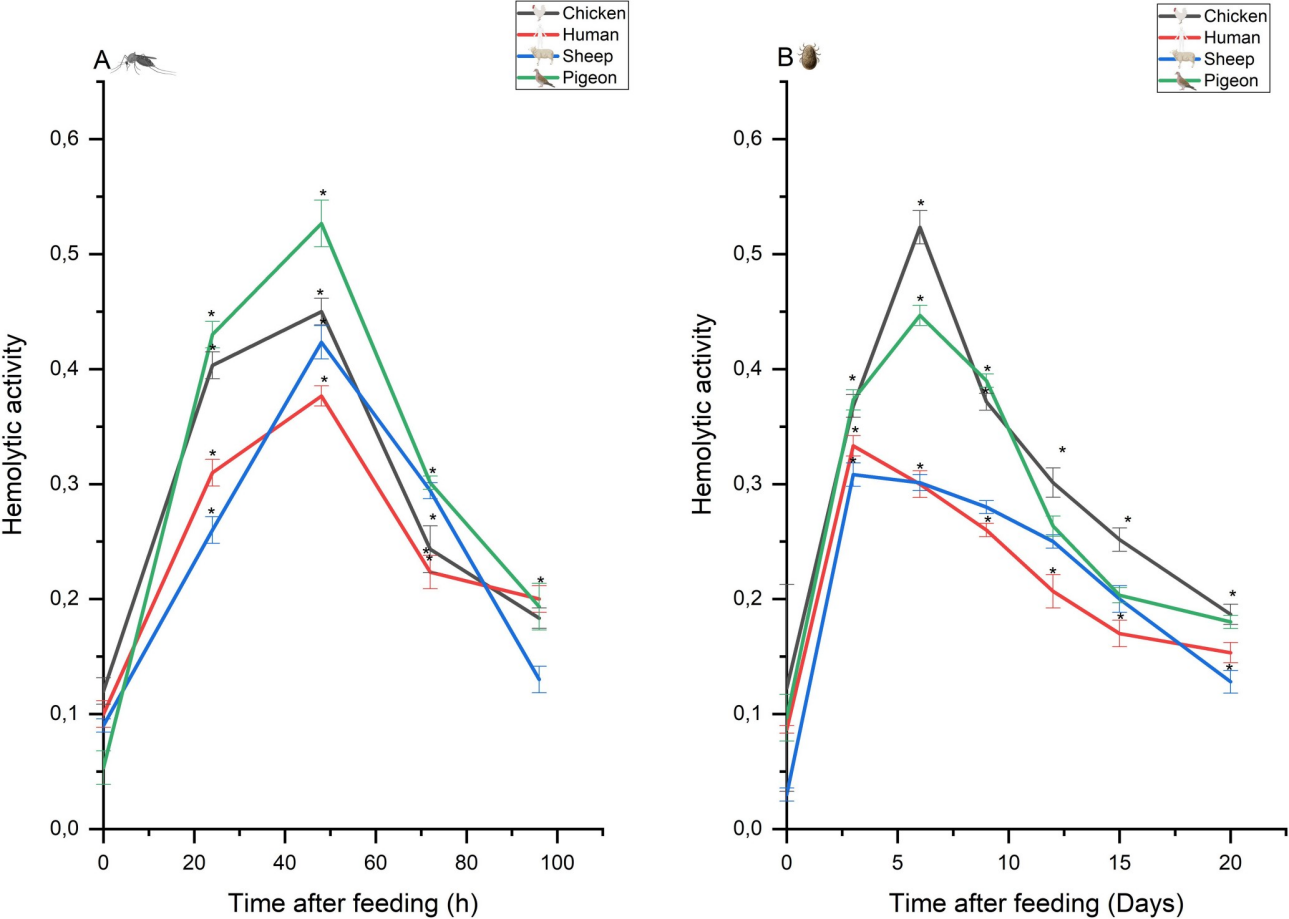
Hemolytic activity in female *Culex pipiens* (A) and *Argas persicus* (B) after different intervals after ingestion of a blood meal from different hosts (using the RBCs of the same host: chicken, human, pigeon, sheep). Hemolytic activity (±S.E.) is expressed as the absorbance at 418 nm/2 h/mg protein (n = 3). Data points with * symbols are significantly different at *p* < 0.01.

To further investigate the possible selectivity of soft tick and mosquito hemolysins for red blood cells from specific hosts, we next examined erythrocytes from chickens, pigeon, sheep, and humans (Fig 4A and 4B). Unlike the mosquito hemolysis, which peaked at 50 hours post feeding without apparent host specificity (Fig 4A), the *A*. *persicus* hemolytic mechanism peaks much later at ~ 3–6 days post feeding and appears selective for chicken erythrocytes, indicating the presence of enzymes selective for avian erythrocyte membrane components (Fig 4B). However, the possibility of a higher affinity of soft tick hemolysins to soft tick receptors [69] or non-specific increased susceptibility of avian erythrocytes to soft tick hemolytic mechanisms cannot be excluded [68]. Sphingomyelin content also requires consideration, since erythrocytes with low sphingomyelin content have been shown to be more sensitive to lysis by stable fly *S*. *calcitrans* gut homogenates compared to those with high sphingomyelin content [44]. As discussed previously [68], unless the hemolysis rate becomes rate-limiting in tick digestion, species differences may have little physiological importance.

**Fig 5:**
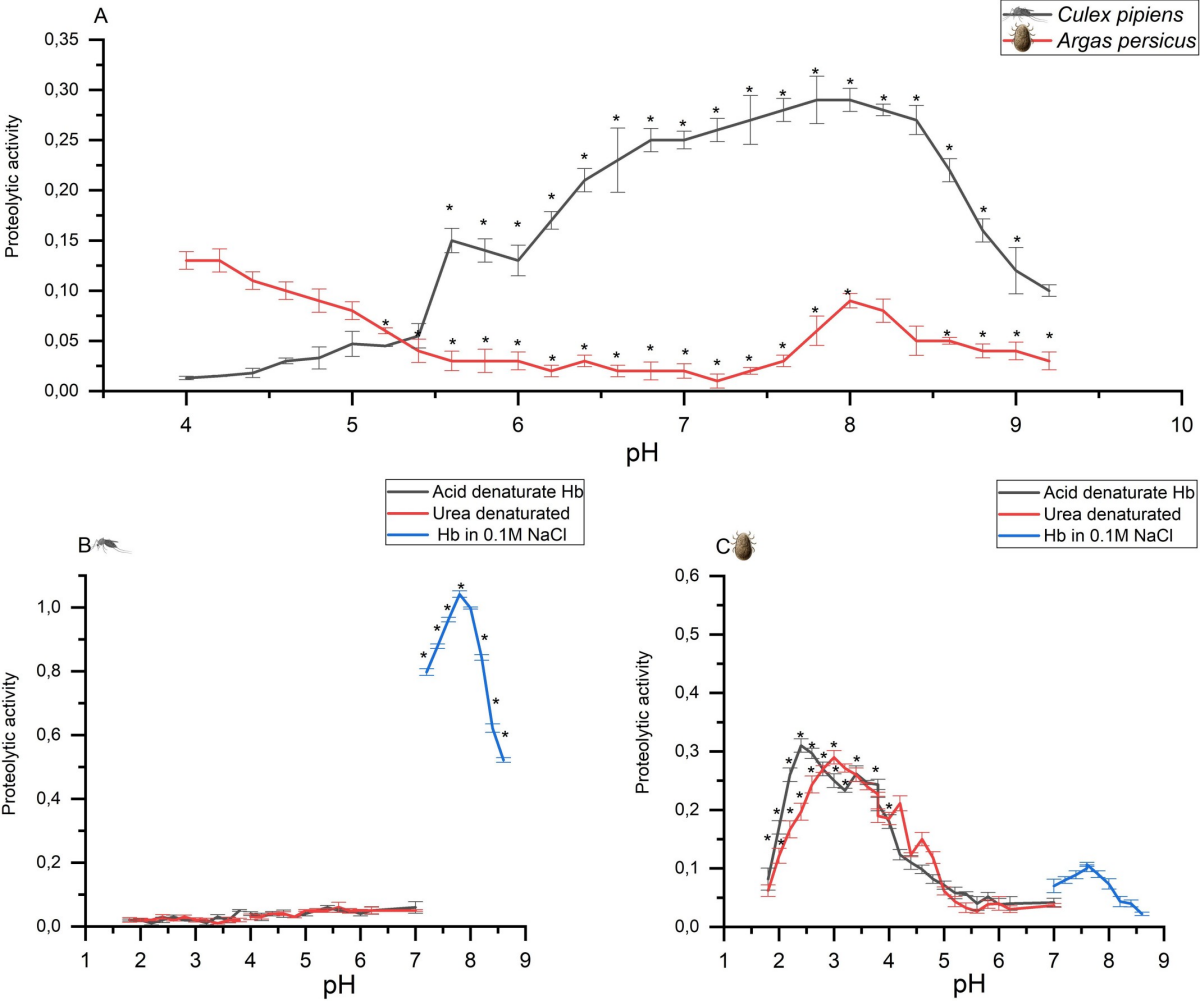
The effect of pH on proteolytic activity (±S.E.). (A) The effect of pH on overall proteolytic activity of homogenates of female *Culex pipiens* and *Argas persicus* using azocasein as a substrate. Expressed as absorbance at 428 nm/h/mg protein. (B) The effect of pH on proteolytic activity of female *Culex pipiens* homogenates using hemoglobin as a substrate. Expressed as absorbance at 280 nm/h/mg protein. (C) The effect of pH on proteolytic activity of female *Argas persicus* homogenates using hemoglobin as a substrate. Proteolytic activity is expressed as absorbance at 280 nm/h/mg protein (n = 3). *: significantly different (*p* < 0.05).

Overall, our results indicate that there is enzymatic (protease and/or phospholipase-based) hemolysis in the gut of the soft tick *A*. *persicus*. The calcium-dependent secreted tick phospholipase A2 was elegantly excluded from this process in the hard tick *I*. *dammini*, as phospholipase A2 requires calcium [70] and *I*. *dammini* hemolytic activity was inhibited by calcium and not modified by EDTA [68]. Other enzymes that might mediate hemolysis are specific proteases that might not digest host blood proteins but specifically lyse host erythrocytes. Therefore, we examined overall gut proteolysis in *A*. *persicus* and *C*. *pipens* at a range of pHs (4–9.5) with azocasein as a substrate (Fig 5A). Consistent with the previous findings in ticks (reviewed in [71]) and mosquitoes (reviewed in [16]), most of the intestinal proteolysis of soluble, not denatured, hemoglobin occurred at neutral and slightly alkaline pHs in mosquitoes (Fig 5B), while in the soft tick massive proteolysis occurs at the acidic pH of endolysosomes [24] (Fig 5C). However, a very important finding has been made: both assay substrates also resulted in a "second" peak in intestinal proteolytic activity at slightly alkaline conditions (pH 7.5–8.0 in Fig 5A and 5C) in the gut homogenates of *A*. *persicus*, indicating possible serine protease activity, similar to that observed in the hard tick *I*. *dammini* [68]. Apparently, tick digestion and hemolysis occur in separate compartments: while blood protein digestion is localized to acidic gut cell endolysosomes [72], alkaline proteolytic hemolysis appears to occur in the neutral conditions of the tick gut lumen. We propose that soft ticks secrete non-digestive serine proteases into the gut lumen that specifically hemolyze host red blood cells. This is consistent with results from the hard tick *Heamaphysalis longicornis*, which secretes a neutral and alkaline hemolytic midgut serine proteinase, HlSP [48]. The respective *A*. *periscus* enzyme(s) now need to be identified and further characterized with respect to their selectivity for chicken hemocytes, as their selective inhibition could inhibit digestion and remove a heme source for *A*. *persicus* as an effective therapeutic strategy to combat this economically important avian ectoparasite and vector of many important diseases.

### How does blood digestion impact pathogen transmission?—perspectives and future trends

In ticks, the fate of ingested pathogens and other microflora in the gut is indirectly linked tightly with blood digestion, albeit ingested microbes are not directly subjected to the intracellularly located digestive enzymes [71]. Hemoglobin-derived hemocidins and other antimicrobial factors and protease inhibitors (reviewed in [72]) are a host defense repertoire that pathogens have to resist in addition to the reactive oxygen species (ROS) generated as a by-product of heme release, causing oxidative stress [27,73]. Similarly, mosquito immunity and their adaptations to hematophagia, gut microbiota, and host blood-derived antimicrobials and inhibitors strongly influence vector competence for human pathogens [74]. However, ticks have developed several protective mechanisms against heme and iron toxicity from host blood. These include the absence of a heme synthetic pathway [75,76], a specialized iron metabolism [77], and heme sequestration in hemosomes [26,77]. In the mosquito *An*. *gambiae*, the balance between ROS and the midgut free radical scavenging enzymes determines the vector competence to transmit malaria [78,79]. Similarly, ticks evidently maintain redox homeostasis within their gut using various antioxidant enzymes and ROS scavengers [80]. Hence, comprehensive research themes on how redox homeostasis is maintained in the mosquito and tick guts, and its impact on the inter-relationships between commensal microflora and transmitted pathogens, may prove fruitful for a better understanding of their vector competence.

### Conclusions, with some caveats

The hemolytic processes in *C*. *pipiens* and *A*. *persicus* are induced in the gut lumen and altered by physiological conditions using distinctive mechanisms. The hemolysis in *C*. *pipiens* is mediated in lipoid fraction by non-enzymatic hemolytic factors in slightly alkali condition while that in *A*. *persicus* involves enzymatic factors in aqueous and acidic environment. While pigeons, chicken, sheep, and human erythrocytes are susceptible for mosquito hemolysis, chicken red blood cells are higher selective for tick digestive mechanism. These studies improve our understanding of initial blood digestion in both species and basic biology of hematophagous arthropods. Inhibiting hemolysis and digestion could help control vector populations and disease transmission.

We believe that the findings of the current study support previous studies demonstrating the requisites of vector’s blood lysis to guarantee an efficient blood digestion; however, we acknowledge that it has limitations. These may include: (1) The lack of data monitoring possible role of midgut flora in blood digestion. However, gene sequencing to study the midgut microorganisms’ communities (metagenomics) remains technically demanding, time consuming, and expensive. To circumvent the potential limitations of microbiological techniques (e.g., autoclaving rearing medium of larval mosquitoes and subculturing of arthropods’ midgut preparations), use of antibiotics potentially affecting arthropods physiology [49] (avoided in this study), and molecular methods, the development of an innovative, robust method that is rapid, cost effective, and reliable in identifying microflora of arthropods’ midguts is required. (2) We performed the different assays in absence of an experimental infection, albeit this is a common scheme in many previous reports. We are planning to expand these experiments to compare results in experimentally-infected arthropods or nutritive blood vs. those in non-infected to determine how infection status (pathogens) may impact blood digestion. (3) The structural features of hemolysins involved in blood digestion processes still need to be elucidated; however, there are currently no works deal with the structural and chemical aspects of these hemolysins, to the best of our knowledge.
